# Advances in gut microbiota research among pediatric patients with sleep-disordered breathing

**DOI:** 10.3389/fmicb.2026.1871503

**Published:** 2026-08-06

**Authors:** Huijie Zhou, Haochen Li, Kailu Zhang, Rongfei Li, Jing Wang, Lei Pan

**Affiliations:** Department of Respiratory and Critical Care Medicine, Shandong Medical and Pharmaceutical University Hospital, Binzhou, China

**Keywords:** gut microbiota, gut-brain axis, intermittent hypoxia, neurocognition, pediatric patients, sleep-disordered breathing

## Abstract

Sleep-disordered breathing (SDB) in children encompasses a spectrum of disorders characterized by abnormal breathing during sleep, with obstructive sleep apnea (OSA) being the most prevalent phenotype. Traditionally, SDB has been viewed as a disorder involving local upper airway structural and neuromodulatory abnormalities. In recent years, the role of the gut microbiota as the “second brain” and a systemic regulator of immunity and metabolism has gained increasing prominence. Accumulating evidence suggests a bidirectional association between pediatric SDB and gut microbiota dysbiosis. This review summarizes recent advances in research on the interplay between pediatric SDB and the gut microbiota, explores the underlying mechanisms—particularly those governing gut microbiota-mediated cognitive and behavioral alterations—and discusses potential diagnostic biomarkers and novel adjunctive therapeutic strategies targeting the gut microbiota. It aims to provide new perspectives for the comprehensive management of pediatric SDB.

## 1 Introduction

Pediatric sleep-disordered breathing (SDB) represents a continuum of upper-airway dysfunction during sleep, ranging from primary snoring and increased respiratory effort to obstructive hypoventilation and obstructive sleep apnea (OSA), with the severity determined by the extent of intermittent upper-airway obstruction (Garde et al., 2022). Its primary etiology involves adenoid and tonsillar hypertrophy (Gulotta et al., 2019), while obesity is emerging as a common risk factor (Qin et al., 2025). Research indicates that the prevalence of OSA in preschool children has risen over the past decade. Recent studies published between 2016 and 2023, after excluding outliers, reported the prevalence of childhood OSA increasing to 12.8%−20.4% (Magnusdottir and Hill, 2024). Pediatric SDB not only causes nocturnal snoring, apnea, intermittent hypoxia (IH), and sleep fragmentation (SF), but is also associated with a range of severe daytime complications. It may further disrupt the programmed developmental trajectory of brain structure and function, leading to long-term cognitive, behavioral, and metabolic consequences (Lo Bue et al., 2020; Liu et al., 2020). Tonsillectomy and adenoidectomy constitute the primary treatment approach, though they are not suitable or effective for all pediatric patients. Moreover, the long-term systemic implications of SDB warrant significant attention (Chuang et al., 2024; Huang et al., 2023; Gulotta et al., 2019).

The gut is often referred to as the “second brain” because of its bidirectional communication with the brain through immune signaling, tryptophan metabolism, the vagus nerve, and the enteric nervous system (Gershon, 1999; O'Connor et al., 2020). The human gut harbors trillions of microorganisms collectively termed the gut microbiota, and dysbiosis has been identified as one complication of OSA (Ko et al., 2019). The gut microbiota is linked to the host physiological and pathological mechanisms (Thursby and Juge, 2017; Matenchuk et al., 2020). However, few studies have directly examined the impact of SDB or OSA on the pediatric gut microbiota. The relationship between SDB-induced gut microbiota changes and the pathogenesis of OSA-related diseases in children remains unconfirmed. Alterations in the gut microbiome are associated with multiple childhood cognitive-behavioral disorders, including anxiety disorders, autism spectrum disorder, attention deficit hyperactivity disorder, and obesity (Checa-Ros et al., 2021; Su et al., 2024; Wan et al., 2022), which bear strong similarities to SDB complications.

Accordingly, we propose that the gut microbiota may contribute to the development of SDB-associated cognitive impairments in children. This review examines gut microbiota alterations in pediatric patients with SDB, the mechanisms that may link SDB to microbial dysbiosis, and the possible contribution of microbiota-related pathways to cognitive and behavioral outcomes. We specifically highlight pediatric-specific microbial features, developmental vulnerability of the pediatric brain, differences between pediatric and adult OSA-associated microbiota profiles, and the translational potential and current limitations of microbiota-based biomarkers and interventions. It aims to provide new perspectives for the comprehensive management of childhood SDB.

## 2 Association between gut microbiota and pediatric SDB

Children with SDB exhibit alterations in gut microbiota abundance and diversity. Studies have reported heterogeneous changes in α-diversity, rather than a uniform increase. In pediatric SDB cohorts, recent polysomnography (PSG)-stratified studies have suggested severity-related changes in specific richness indices and β-diversity (Yang et al., 2024; Maiming et al., 2024; Valentini et al., 2020; Collado et al., 2019; Wang et al., 2025; Chen et al., 2026). At the phylum level, Firmicutes, Bacteroidetes, Actinobacteria, and Proteobacteria were the most dominant phyla across all samples. Firmicutes and Proteobacteria constituted the dominant microbiota in pediatric SDB patients, while Bacteroidetes was predominant in healthy individuals (Yang et al., 2024). Research data further confirms that the relative abundance ratio of Firmicutes to Bacteroidetes (F/B) is higher in pediatric SDB patients than in controls (Maiming et al., 2024; Collado et al., 2019). However, the F/B ratio is not a stable or universally reliable marker of dysbiosis, and should be considered only a coarse descriptive index rather than a deterministic biomarker for pediatric SDB/OSA. Wang et al. (2025) included a total of 124 Chinese patients aged between 2 and 16 years old (62 with OSA; 16 with primary snoring; 46 controls) and found that Firmicutes abundance and F/B showed only limited diagnostic performance, with area under the curve (AUC) values only slightly above 0.50. At the genus level, pediatric SDB patients exhibited relative increases in Muribaculaceae, *Klebsiella, Eubacterium, Faecalibacterium*, unclassified Enterobacteriaceae, and *Roseburia*, alongside relative decreases in *Bacteroides* and unclassified Ruminococcaceae (Yang et al., 2024; Wu et al., 2022). Chen et al. (2026) found *Faecalibacterium* and *Bacteroides* enriched in snoring children but *Bifidobacterium* and *Akkermansia* enriched in controls. Furthermore, statistically significant differences were observed between sleep quality and obstructive sleep apnea hypopnea index (OAHI) and relative microbial abundance. At the phylum level, sleep quality showed a positive correlation with the Bacteroidetes phylum. At the genus level, sleep quality was negatively correlated with *Bifidobacterium* and *Lactobacillus*, and positively correlated with *Bacteroides*. The OAHI was negatively correlated with Enterobacter (Maiming et al., 2024). Adult OSA patients, conversely, exhibit elevated levels of Bifidobacteria, yet reduced levels of *Faecalibacterium, Megamonas, Ruminococcaceae, Clostridiales*, and *Alistipes* compared to non-OSA individuals (Ko et al., 2019). This may be related to differences in taxonomic-level findings across studies and may be influenced by factors such as age, geographic region, obesity or weight status, diet, disease definition and severity, sample size, sequencing platform, targeted 16S rRNA region, bioinformatics workflow, and fecal sampling or storage methods. Multiple prior studies have demonstrated significant alterations in the composition and abundance of gut microbiota in pediatric SDB patients (Table 1). It should be noted that taxonomic signatures alone are insufficient as robust diagnostic biomarkers due to high inter-individual variability, functional redundancy, and limited cross-cohort reproducibility; therefore, their diagnostic utility remains constrained. The gut microbiota of pediatric SDB patients is characterized by reduced diversity and abundance of beneficial flora, demonstrating significant correlations with metabolic pathways. These findings underscore the necessity for further exploration of the role, mechanisms, and therapeutic potential of the gut microbiota, particularly in pediatrics, to better comprehend its broad implications for health management and long-term disease outcomes.

**Table 1:** Summary of gut flora characteristics of children with SDB.

References	Object (age)	The type of insomnia/design	Sample size (experimental group)	The sequencing methods	Country	Classification	Ascend	Descend	Predominant bacteria
Yang et al. (2024)	Patient (2–12y)	Non-obese children with enlarged tonsils and adenoids	63 (47)	16S rRNA	China	Phylum	*Proteobacteria*	*Bacteroidetes*	*Firmicutes, Bacteroidetes, Actinobacteria, Proteobacteria*
						Order	*Lactobacillales, Enterobacterales*	*Bacteroidales, Lachnospirales, Bifidobacteriales*	
						Genus	*genus Muribaculaceae, Klebsiella*	*genus Bacteroides*	
Maiming et al. (2024)	Patient(3–6y)	Non-obese children with OSA	60 (30)	16S rRNA	China	Phylum	*Firmicutes*	*Bacteroidetes*	
Collado et al. (2019)	Patient(2y)	Non-obese children with snorers	43 (27)	16S rRNA	Finland	Phylum	*Proteobacteria*		
						Family	*Enterobacteriaceae, Erysipelotrichaceae*	*Ruminococcaceae, unclassified Clostridiales*	
						Genus	*unclassified Enterobacteriaceae, Eubacterium*		
Wu et al. (2022)	Patient (4–6y)	Non-obese children with OSA	88 (43)	16S rRNA	China	Genus	*Faecalibacterium, Roseburia*	*unclassified Ruminococcaceae*	*Bacteroides, Bifidobacterium, Faecalibacterium, Blautia*
Valentini et al. (2020)	Patient (2–12y)	Non-obese children with OSA	16 (7)	16S rRNA	Italy	Genus	*unclassified Enterobacteriaceae, Eubacterium*		
						Family	*Clostridiaceae, Oscillospiraceae, Enterobacteriaceae, Proteobacteria, Desulfovibrionaceae*	*Lactobacillaceae, Coriobacteriaceae*	
						Genus	*Escherichia, Eubacterium, Klebsiella, Clostridium*		
Wang et al. (2025)	Patient (2–16y)	Children with primary snoring/OSA	124 (62 OSA; 16 primary snoring)	16S rRNA	China	Phylum	*Firmicutes, Verrucomicrobia*		*Firmicutes, Actinobacteriota, Bacteroidota, Proteobacteria, Verrucomicrobia*
						Genus	*Akkermansia*	*Bacteroides, Escherichia-Shigella, Parabacteroides*	*Bifidobacterium, Bacteroides, Faecalibacterium, Escherichia-Shigella, Streptococcus*
Chen et al. (2026)	Patient (2–9y)	Children with snoring	60 (30)	16S rRNA	China	Genus	*Faecalibacterium, Bacteroides*	*Bifidobacterium, Akkermansia*	*Faecalibacterium, Bacteroides, Bifidobacterium, Akkermansia*

Classification of biodiversity: Kingdom, Phylum, Class, Order, Family, Genus, Species.

## 3 Mechanisms linking pediatric SDB to gut microbiota dysbiosis

**Figure 1:**
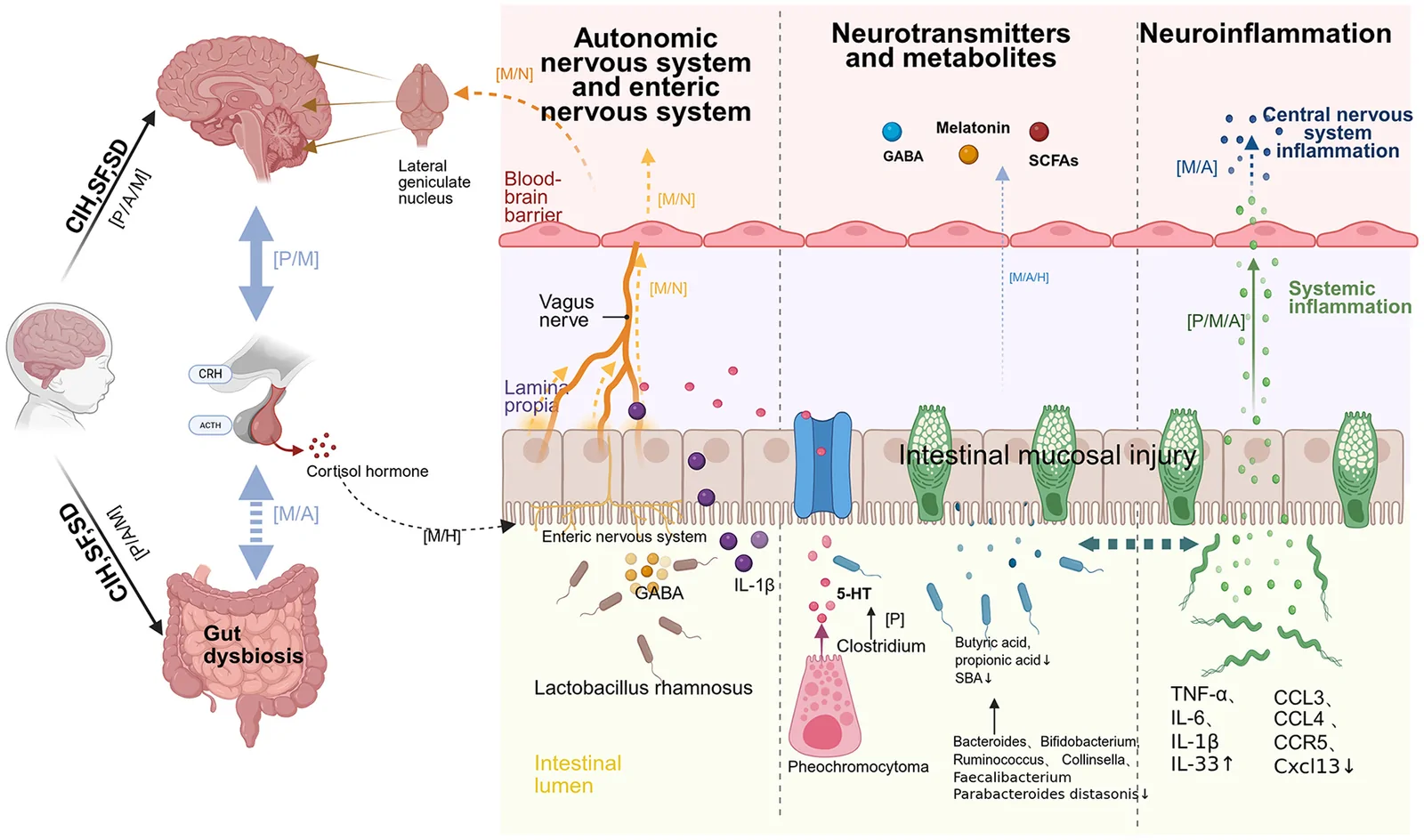
Evidence-graded pathways linking gut microbiota and pediatric sleep-disordered breathing interactions. SDB, especially OSA, may interact with gut microbiota through intermittent hypoxia, sleep fragmentation, neuroendocrine dysregulation, systemic inflammation, intestinal barrier injury, and microbial metabolic alterations. Conversely, gut dysbiosis may contribute to SDB-related systemic complications through inflammatory signaling, LPS/TLR4/NF-κB activation, impaired intestinal barrier integrity, and altered microbial metabolites. Solid arrows indicate direct pediatric SDB/OSA evidence; dashed arrows indicate indirect evidence from adult OSA, animal, or *in vitro* studies; dotted arrows indicate hypothetical or unvalidated pathways. [P], pediatric SDB/OSA; [A], adult OSA; [M], animal or *in vitro* model; [N], non-SDB pediatric neurodevelopmental disorder; and [H], hypothesis. SDB, sleep-disordered breathing; OSA, obstructive sleep apnea; IH, intermittent hypoxia; SF, sleep fragmentation; SD, sleep deprivation/ sleep disruption; HPA axis, hypothalamic-pituitary-adrenal axis; LPS, lipopolysaccharide; TLR4, Toll-like receptor 4; NF-κB, nuclear factor kappa-B; TNF-α, tumor necrosis factor-alpha; IL-6, interleukin-6; IL-17, interleukin-17; IL-1β, interleukin-1 beta; IL-33, interleukin-33; D-LA, D-lactic acid; I-FABP, intestinal fatty acid-binding protein; TJ, tight junction; ZO-1, zonula occludens-1; sIgA, secretory immunoglobulin A; SCFAs, short-chain fatty acids; CCL3, C-C motif chemokine ligand 3; CCL4, C-C motif chemokine ligand 4; CCR5, C-C chemokine receptor type 5. Created with BioRender.com.

SDB progresses from simple snoring to OSA, with OSA representing the most severe clinical manifestation of pediatric SDB. It primarily involves chronic IH, SF and sleep deprivation (SD). SDB may influence the gut microbiota through multiple pathways, as illustrated in (Figure 1).

### 3.1 Effects of IH on the gut microbiota

In children with OSA, IH may elicit ischemia-reperfusion injury in the intestinal mucosa. Inadequate oxygen delivery to the mucosal tissue may further trigger changes in both the composition and abundance of the gut microbiota (Valentini et al., 2020; Li and Shi, 2023). The IH/reoxygenation cycle mimics ischemia-reperfusion injury, generating reactive oxygen species (ROS) and activating inflammatory signaling cascades including nuclear factor kappa-B (NF-κB), thereby leading to systemic low-grade inflammation (Dong et al., 2024). Inflammatory mediators, such as tumor necrosis factor-alpha (TNF-α) and interleukin (IL)-6, affect intestinal barrier integrity via the circulatory system, altering the gut microenvironment. This inhibits beneficial bacteria (such as short-chain fatty acid-producing microbiota) while promoting the proliferation of opportunistic pathogens (Valentini et al., 2020; Lv et al., 2023). Evidence from animal models further supports a role for IH in intestinal inflammation and barrier dysfunction. Studies demonstrate that in two experimental models—C57BL/6 mice exposed to IH for 6 weeks and rats exposed to IH for 28 h, the levels of typical pro-inflammatory cytokines in the serum were significantly elevated (Li and Shi, 2023; Zhang et al., 2022). IH also promotes T helper 17 (Th17) differentiation by enhancing phosphorylation of signal transducer and activator of transcription 3 (STAT3) *in vitro*, thereby elevating pro-inflammatory cytokines in circulation (Li et al., 2023b). In addition, microbial abundance in adult OSA patients was correlated with inflammatory cytokine levels (Liu et al., 2024a). This suggests that gut microbiota dysbiosis in OSA patients may be associated with systemic chronic inflammation.

IH may also alter the intestinal oxygen environment. IH induces periodic decreases in intestinal oxygen partial pressure during sleep, leading to alterations in the relative abundance of aerobic bacteria and increases in the relative abundance of facultative and obligate anaerobes (Li and Shi, 2023). Increased anaerobic bacterial load may compromise the integrity of tight junctions in intestinal epithelial cells, thereby increasing intestinal mucosal permeability and facilitating bacterial translocation. Such gut barrier disruption may subsequently influence the gut microbiota (Li et al., 2023a). Li et al. (2021) reported that plasma levels of intestinal mucosal injury markers D-lactic acid (D-LA) and intestinal fatty acid-binding protein (I-FABP) were significantly elevated in adults with OSA. Consistent with this observation, experiments confirmed that IH induced a significant reduction in serum expression of tight junction (TJ) proteins and related occludin, claudin-1/5, and zonula occludens-1 (ZO-1) in mice (Xue et al., 2025; Li et al., 2023b). Further studies indicate that *Faecalibacterium* can restore intestinal barrier structure and function by modulating tight junction pathways and ZO-1 expression (Xu et al., 2020). These findings suggest hypoxia induced by OSA may damage the intestinal barrier. In addition to inflammatory and barrier-related effects, IH may influence metabolic and endocrine regulation. IH can influence hormone levels such as leptin and cortisol, and jointly participates with the gut microbiota in regulating host energy metabolism (Dong et al., 2024). In summary, IH primarily affects gut microbiome balance through the oxidative stress-inflammation axis, the intestinal barrier damage axis, and the metabolic-endocrine axis.

### 3.2 Effects of SF and SD on gut microbiota

Numerous animal studies have shown that SF and SD can lead to gut microbial dysbiosis through multiple mechanisms, providing indirect evidence for microbiota disruption in pediatric SDB. In Sprague Dawley rats, SF and SD directly act on the central nervous system and trigger stress responses involving the hypothalamic-pituitary-adrenal (HPA) axis (Triplett et al., 2020). Glucocorticoid levels were abnormally elevated in some SD and SF animal models. Elevated cortisol influences the gut via the circulatory system, altering intestinal motility, secretory function, and permeability, while directly inhibiting the growth of beneficial commensal bacteria, such as *Lactobacilli* and *Bifidobacteria*, thereby creating conditions conducive to the colonization of potential pathogenic bacteria (Matenchuk et al., 2020). However, it is notable that in the study by Triplett et al., it was observed that the level of adrenocorticotropic hormone (ACTH) decreased in the rat model, while glucocorticoids did not increase. This suggests that SD and SF can perturb HPA axis activity, but their effects on glucocorticoids are dependent on the sampling window and experimental paradigm. SD and IH trigger multiple inflammatory pathways, exacerbating tissue responses to reactive oxygen species release and oxidative stress. This leads to a reduction in oxygen supply to the intestinal mucosa and overall damage to the intestinal barrier (Da Silva et al., 2024; Zhang et al., 2025). Lai et al. (2022) corroborated these findings, demonstrating reduced goblet cell numbers in SD rats alongside increased expression of select tight junction proteins in both gut and brain tissues. In the C57BL/6J mouse model, chronic SD altered the gut microbiota and induced systemic inflammation, adipose tissue inflammation and insulin resistance, subsequently affecting the microbiota. At the same time, IH and SF can induce increased food intake, while chronic SD can alter energy metabolism (Poroyko et al., 2016; Wang et al., 2022). Such dietary structural alterations directly provide gut microbes with distinct fermentation substrates, rapidly reshaping microbial composition and promoting the proliferation of pro-inflammatory bacterial populations. In synergy with hypoxic effects, SDB-associated IH and SF may lead to persistently heightened sympathetic nervous system excitability coupled with relatively insufficient vagal tone (Olivares et al., 2022). The release of catecholamine hormones directly or indirectly impacts intestinal motility, secretion, and permeability, disrupting microbial balance by regulating bacterial virulence gene expression (Brown, 2016). Chronic SD caused by SDB can disturb the circadian rhythms of intestinal epithelial cells and the gut microbiota itself. The composition and metabolic activity of the gut microbiota exhibit significant diurnal oscillations. Disruption of host circadian genes can directly impair this microbial rhythm by altering the intestinal environment, leading to microbial dysbiosis (Lv et al., 2023). In pediatric SDB, SF and SD may profoundly disrupt the stability of the gut microbial ecosystem through multiple pathways—either independently or synergistically with IH—by activating the neuroendocrine stress axis, causing autonomic imbalance, disrupting the host circadian clock, and inducing adverse behavioral and metabolic alterations.

### 3.3 Potential contribution of gut microbiota to SDB development and progression

Dysbiotic gut microbiota should not be regarded merely as a passive consequence of SDB. It may exert bidirectional effects on the systemic pathways and potentially exacerbate the pathological process of SDB. Mendelian randomization studies have suggested genetically predicted associations between specific microbial taxa, microbial metabolites, and OSA risk (Wei et al., 2023; Yan et al., 2023). Regarding direct population evidence, existing studies suggest associations between gut microbiota alterations and inflammatory, metabolic, and cardiovascular-related phenotypes. Huang et al. (2024) enrolled 60 children aged 5–12 years with OSA from Taiwan. The study found that β-diversity of the gut microbiota and 31 marker taxa were associated with blood pressure changes, with Acinetobacter linked to IL-17/TNF-α and blood pressure fluctuations, indicating a potential “microbiota alteration-inflammation-abnormal blood pressure” pathway in pediatric OSA.

Animal models provide further mechanistic support. Ayyaswamy et al. found that, in an 8-week-old male rat model of OSA-induced hypertension, gut microbiota dysbiosis induced pro-inflammatory responses in both the gut and brain through IL-17A signaling (Ayyaswamy et al., 2023). Another large mouse experiment suggested that gut dysbiosis influences respiratory center and muscle function via the gut-brain axis. Microbiota metabolites and inflammatory signals may disrupt brainstem respiratory center regulation and potentially affect upper-airway dilator muscle tone (Li and Shi, 2023). In parallel, inflammatory signaling within adenotonsillar tissues may contribute to lymphoid tissue proliferation and upper-airway obstruction in pediatric OSA. Adenotonsillar hypertrophy is a major anatomical contributor to pediatric OSA, and studies of adenotonsillar tissues from children with OSA have shown increased cellular proliferation and elevated pro-inflammatory mediators, including TNF-α, IL-6, and IL-1α (Kim et al., 2009). Leukotriene-related pathways may also promote adenotonsillar inflammatory and proliferative responses, as leukotriene stimulation increases adenotonsillar cell proliferation *in vitro*, whereas leukotriene pathway inhibition reduces proliferation and cytokine production (Dayyat et al., 2009). These findings suggest a potential feed-forward loop in which upper-airway inflammation and lymphoid tissue hypertrophy reinforce airway obstruction. Persistent systemic inflammation may chronically stimulate lymphoid tissues in the nasopharynx and oropharynx—including the adenoids and palatine tonsils—driving pathological lymphoid hyperplasia and thereby establishing a self-perpetuating “inflammation-hyperplasia-worsening upper airway obstruction” cycle. However, whether gut microbiota-driven systemic inflammation disrupts brainstem respiratory center regulation and directly promotes adenotonsillar hypertrophy in children with SDB remains unproven. Fecal microbiota transplantation from C57BL/6J mice exposed to IH induces sleep disturbances in germ-free recipients (Badran et al., 2020). This further indicates that the microbiota changes induced by IH have the ability to affect sleep phenotypes.

Gut microbial dysbiosis may also contribute to SDB complications through intestinal barrier damage and endotoxemia. Dysbiosis is characterized by overgrowth of pathogenic bacteria and depletion of beneficial flora, thereby increasing the production of intestinal toxins, such as lipopolysaccharide (LPS) (Moreno-Indias et al., 2016). LPS mediates inflammation and impairs intestinal barrier function through the LPS/Toll-like receptor 4 (TLR4)/NF-κB pathway. Endotoxins may translocate into the bloodstream through the compromised intestinal barrier, directly affecting vascular endothelial cells and inducing metabolic endotoxemia (Kalyan et al., 2022). Concurrently, activation of TLR4 releases pro-inflammatory cytokines, triggering low-grade systemic inflammation that further induces inflammation and oxidative stress (Liu et al., 2022), leading to SDB complications including hypertension and cognitive or behavioral alterations (Yin et al., 2024; Zhang et al., 2025). Moreno-Indias et al. found in the IH model of C57BL/6 mice that the dysbiosis and low-grade endotoxemia induced by IH were not completely reversed after normoxia recovery (Moreno-Indias et al., 2016). This animal evidence suggests that the changes related to the microbiota and endotoxins after hypoxia relief may be persistent, but their reversibility after the treatment of SDB in human children still requires longitudinal cohort verification. In adult OSA, alterations in the gut microbiota have been found to be associated with metabolic disorders. Ko et al. studied 93 adult Chinese patients with OSA and 20 non-OSA controls. The research report states that microbiome alterations in patients with OSA are associated with metabolic comorbidities, including correlations with homocysteine levels (Ko et al., 2019). However, since this evidence comes from adult groups other than animals rather than children, it can only serve as supporting evidence for systemic metabolic disorders related to OSA, but not as direct evidence of SDB in children.

Overall, current evidence suggests that gut microbial dysbiosis may participate in SDB-related pathological processes and systemic complications through immune, metabolic, and neural regulatory pathways.

## 4 Microbiota-related pathways potentially linking pediatric SDB to cognitive and behavioral alterations

OSA impairs cognitive function, with more pronounced effects observed in pediatric patients (Lv et al., 2024). During untreated apneas and hypopneas, both adults and children experience IH, reoxygenation, and hypercapnia or hypocapnia, accompanied by alterations in cerebral blood flow and fragmented sleep. These effects may lead to cognitive deficits impairing work and learning efficiency. Such neurocognitive impairments correlate with age, being more pronounced in older children (Tamanyan et al., 2018). Similarly, Menzies et al. (2022) reported that neurocognitive deficits are present across all severity levels of SDB in children, though the severity of SDB does not consistently correlate with more severe or widespread neurocognitive deficits. Pediatric SDB is associated with acute cerebral changes affecting autonomic regulation, cognitive and emotional functions, alongside chronic alterations in the prefrontal cortex critical for behavioral control (Horne et al., 2018). Substantial evidence indicates that the gut microbiota plays a pivotal role in several childhood neuropsychiatric disorders, including autism, depression, anxiety, schizophrenia, and attention deficit/hyperactivity disorder, alongside associated emotional and behavioral symptoms (Bundgaard-Nielsen et al., 2023; Wali et al., 2021). However, few studies have directly investigated the mechanisms by which gut microbiota mediate cognitive and behavioral alterations in pediatric SDB patients. Therefore, we summarize the mechanisms of gut microbiota in cognitive and behavioral changes among adult SDB patients and in children with comorbid cognitive and behavioral alterations.

**Figure 2:**
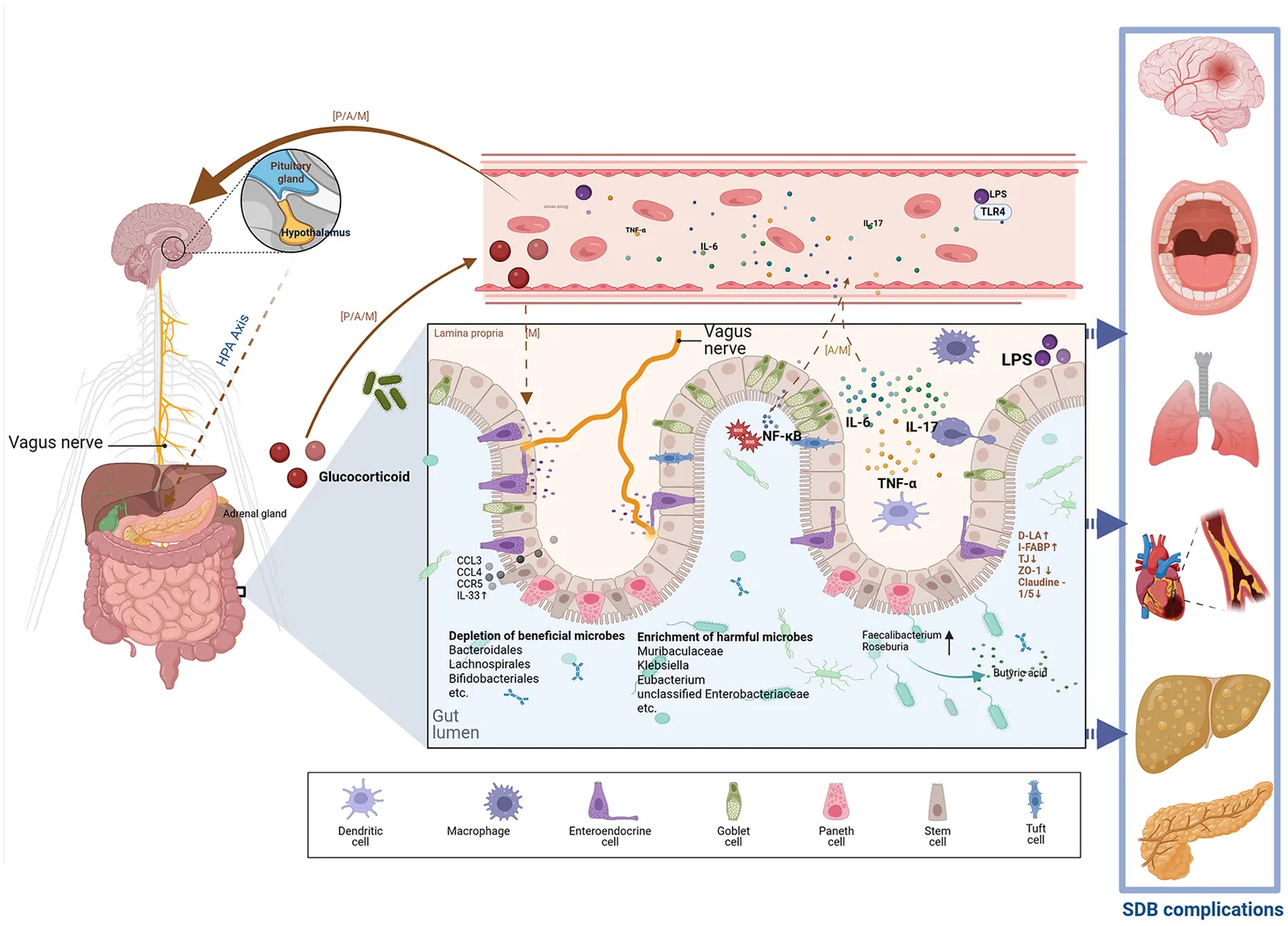
Evidence-graded microbiota-related pathways potentially linking pediatric sleep-disordered breathing to cognitive and behavioral alterations. SDB and OSA are associated with intermittent hypoxia, sleep fragmentation, sleep disruption, gut microbiota dysbiosis, and neurocognitive or behavioral alterations. This figure summarizes potential microbiota-related mechanisms involving the microbiota-gut-brain axis (MGBA), including autonomic and enteric nervous system signaling, hypothalamic-pituitary-adrenal (HPA)-axis-related stress responses, neurotransmitter and microbial metabolite pathways, intestinal mucosal injury, systemic inflammation, and neuroinflammation. Solid arrows indicate pathways supported by direct pediatric SDB/OSA evidence. Solid arrows indicate direct pediatric SDB/OSA evidence; dashed arrows indicate indirect evidence from adult OSA, animal, or *in vitro* studies; dotted arrows indicate hypothetical or unvalidated pathways. [P], pediatric SDB/OSA; [A], adult OSA; [M], animal or *in vitro* model; [N], non-SDB pediatric neurodevelopmental disorder; and [H], hypothesis. CIH, chronic intermittent hypoxia; SF, sleep fragmentation; SD, sleep deprivation/ sleep disruption; HPA axis, hypothalamic-pituitary-adrenal axis; CRH, corticotropin-releasing hormone; ACTH, adrenocorticotropic hormone; GABA, gamma-aminobutyric acid; 5-HT, 5-hydroxytryptamine; SCFAs, short-chain fatty acids; SBA, secondary bile acids; TNF-α, tumor necrosis factor-alpha; IL-1β, interleukin-1 beta; IL-6, interleukin-6; IL-17, interleukin-17; IL-33, interleukin-33; CCL3, C-C motif chemokine ligand 3; CCL4, C-C motif chemokine ligand 4; CCR5, C-C chemokine receptor type 5; Cxcl13, C-X-C motif chemokine ligand 13. Created with BioRender.com.

Previous studies indicate that signaling within the microbiota-gut-brain axis (MGBA) may play an important role in this process. The MGBA is a bidirectional signaling pathway connecting the gut microbiota with the central nervous system (CNS) and the enteric nervous system (ENS). In pediatric SDB, a balanced and stable gut microbiome under normal physiological conditions can favorably regulate central nervous system function through the MGBA, which is essential for preserving intestinal barrier integrity and controlling inflammatory homeostasis. Under pathological conditions, dysbiosis may contribute to the pathophysiology of cognitive-behavioral impairment through core mechanisms including driving neuroinflammation, disrupting neurotransmitter balance, impairing HPA axis function, and altering key neurotransmitters. Dysbiosis may represent a potential pathway in cognitive deficits arising from sleep disorders. The MGBA is increasingly recognized as a vital physiological mechanism for maintaining health and influencing the brain and central nervous system (O'Connor et al., 2020). Endocrine and metabolic pathways, immune pathways, and neuronal pathways are currently key avenues of investigation (Oroojzadeh et al., 2022). The mechanism by which OSA interacts with the gut microbiota to induce cognitive behavioral disorders is primarily associated with the MGBA, as illustrated in (Figure 2).

The evidence supporting MGBA involvement in pediatric SDB is currently heterogeneous. Direct evidence from pediatric SDB cohorts is limited, whereas much of the mechanistic interpretation is derived from pediatric neurodevelopmental disorders, adult OSA studies, and animal models. Therefore, these indirect findings should be discussed as supporting biological plausibility rather than as definitive evidence of microbiota-mediated mechanisms in children with SDB. The level of evidence for each proposed mechanism is summarized in Table 2, and the characteristics of the different evidence sources are further presented in (Table 3). The available human clinical/cohort and animal evidence is summarized in Table 4.

**Table 2:** Evidence levels for potential microbiota-gut-brain mechanisms in pediatric SDB.

Mechanism	Direct pediatric SDB evidence	Pediatric neurodevelopmental/psychiatric evidence	Adult OSA evidence	Animal evidence evidence	Strength
Gut microbiota alteration in pediatric SDB	Direct but limited evidence; mainly association-based	Supportive evidence from ASD, ADHD, anxiety, and depression studies showing microbiota-behavior associations	Supportive evidence	Limited	Moderate
Intermittent hypoxia and intestinal barrier damage	Limited	Indirect evidence from inflammatory and neurodevelopmental studies	Supportive evidence	Strong	Moderate
SCFA-related mechanisms	Limited	Moderate indirect evidence from neurodevelopmental and psychiatric disorders	Moderate	Strong	Hypothesis-generating
Vagus nerve pathway	Very limited	Limited indirect evidence from microbiota-gut-brain studies	Limited	Strong	Speculative
HPA axis involvement	Limited	Moderate indirect evidence from stress-related, anxiety, and depression studies	Moderate	Moderate	Hypothesis-generating
Microbiota-mediated cognitive impairment	Very limited	Moderate indirect evidence from ASD, ADHD, anxiety, depression, and cognitive studies	Moderate	Moderate	Speculative

**Table 3:** Summary of evidence sources related to MGBA in pediatric SDB and related conditions.

Evidence category	Study population/model	Disease/context	Age/species	Main finding	Relevance to pediatric SDB	Limitation
Direct pediatric evidence	Children	SDB/OSA	Children/adolescents	Neurocognitive or behavioral changes	Directly relevant	Microbiota evidence limited
Pediatric neurodevelopmental evidence	Children	ASD/ADHD/anxiety/depression	Children/adolescents	Gut microbiota associated with behavior/cognition	Indirect support for gut-brain axis	Not SDB-specific
Adult OSA evidence	Adults	OSA	Adults	Intermittent hypoxia associated with dysbiosis/inflammation	Supports possible mechanisms	Cannot be directly extrapolated to children
Animal model evidence	Mice/rats/etc.	Intermittent hypoxia/sleep fragmentation	Animal species	Inflammation, oxidative stress, microbial metabolites affect brain	Mechanistic support	Experimental model only

**Table 4:** Summary of human clinical/cohort and animal studies on sleep-disordered breathing/OSA and gut microbiota.

References	Participants/animal species	Age	Country	Disease severity or experimental model	Sequencing method and bioinformatics	Sampling and key controls
Pediatric human clinical/cohort studies						
Yang et al. (2024)	63 (47 SDB)	2–12 y	China	SDB by SCR/SCS ≥6.5; no PSG severity stratification	High-throughput 16S rRNA; platform NR; V3–V4; primers 343F/798R; QIIME 1.8, LEfSe, PICRUSt/KEGG	Sampling/storage: Morning feces; refrigerated transport; −80 °C within 1 h; Obesity: Non-obese; obesity excluded; Diet: NR; Antibiotic/probiotic: Excluded antibiotics/anti-inflammatory drugs within 2 months and probiotic use
Maiming et al. (2024)	60 (30 OSA)	3–6 y	China	OSAHS diagnosed by Chinese pediatric guideline; OAHI/LSaO_2_/MSaO_2_ recorded; not stratified	NovaSeq 6000 (PE250); 16S V3–V4; QIIME2-DADA2; ASVs at 97%; NMDS/weighted UniFrac; LEfSe; Spearman correlation	Sampling/storage: ~1 g midstream fresh feces; −80 °C within 30 min; Obesity: BMI comparable; obesity status not specifically controlled; Diet: NR; Antibiotic/probiotic: Antibiotics within 1 month excluded; probiotic restriction NR
Valentini et al. (2020)	16 (7 OSA/OSAS)	2–12 y	Italy	PSG-confirmed OSA/OSAS; AHI ≥1; mean AHI 10.8 ± 5.3 events/h	Ion Torrent Ion S5 (Ion 510 chip); Broad 16S hypervariable regions V1–V9 (Ion 16S kit: V2/4/8 and V3/6–7/9); GAIA, PAST, ANOSIM/SIMPER	Sampling/storage: Sterile tube; refrigerated transport; −80 °C within 8 h; Obesity: Obesity excluded; BMI comparable; Diet: Current diet NR; non-breastfed children excluded; Antibiotic/probiotic: Excluded antibiotics within 60 d, probiotics within 30 d, anti-inflammatory drugs within 15 d
Collado et al. (2019)	43 (27 snorers)	24 mo	Finland	Snoring ≥3 nights/week; no PSG severity assessment	Illumina MiSeq 2 × 300; V3–V4; QIIME 1.9/Greengenes 13.8; ANCOM, DESeq2, LEfSe	Sampling/storage: Feces collected at 24 mo; frozen/stored at−80 °C; Obesity: BMI comparable; obesity not used as exclusion; Diet: Breastfeeding duration recorded; current diet not controlled; Antibiotic/probiotic: Antibiotic prescriptions in previous year recorded; probiotic exposure NR
Wu et al. (2022)	88 (43 OSA)	4–6 y	China	PSG-confirmed OSAHS; mean AHI 8.19 ± 5.32 events/h, LaSO2 72.4 ± 15.5%	Illumina MiSeq; V4; primers 515F/806R; Usearch 11, RDP, mothur, R packages, PICRUSt, STAMP	Sampling/storage: Fresh feces; −80 °C immediately after collection; Obesity: Obesity excluded; BMI comparable; Diet: NR; Antibiotic/probiotic: Excluded antibiotics and probiotics within 3 months
Wang et al. (2025)	124 (62 OSAHS; 16 primary snoring; 46 controls)	2–16 y	China	PSG confirmed; 42 mild OSAHS and 20 moderate-to-severe OSAHS; primary snoring group included	Illumina NovaSeq PE250; V3–V4; primers 341F/805R; FLASH, fqtrim, Vsearch, DADA2, SILVA 138, QIIME2, LEfSe, RDA/Spearman, ROC	Sampling/storage: Clean stool ≥100 mg; transferred to −80 °C within 4 h; Obesity: Age/sex/BMI comparable across groups; obesity not specifically excluded; Diet: NR; Antibiotic/probiotic: Excluded antibiotics within 60 d, probiotics within 30 d, anti-inflammatory drugs within 15 d
Chen et al. (2026)	60 (30 snoring; 30 controls)	2–9 y	China	Snoring ≥1 month with rhinitis; no PSG stratification; pre-surgery feces	Illumina NovaSeq 6000 SP, 2 × 250 bp; V3–V4; primers 341F/805R; QIIME2, Cutadapt, FLASH, fqtrim, Vsearch/SILVA, DADA2 ASVs, LEfSe/indicator analysis, PICRUSt2/KEGG; untargeted metabolomics	Sampling/storage: Morning feces within 1 h; snap-frozen in liquid nitrogen and transferred to −80 °C within 2 h; Obesity: BMI comparable; chronic diseases excluded; Diet: NR; Antibiotic/probiotic: Excluded antibiotics/probiotics/anti-inflammatory drugs within 2 weeks
Huang et al. (2024)	60 OSA/OSAS children	5–12 y; median 7 y	Taiwan, China	Cross-sectional OSA/OSAS cohort; AHI assessed by PSG; BP categories analyzed	Illumina MiSeq 2 × 300; V3–V4; primers 341F/806R; FLASH 1.2.11, QIIME 1.9.1, UCHIME, USEARCH 7.0.1090, Silva 132, PyNAST; cytokine profiling	Sampling/storage: Morning stool collected by parents; immediately snap-frozen; stored at −80 °C; Obesity: BMI recorded; craniofacial/neuromuscular/chronic inflammatory disorders including asthma/autoimmune disease excluded; Diet: NR; Antibiotic/probiotic: Antibiotic therapy required ≥2-week remission before sampling; probiotic restriction NR
Chuang et al. (2024)	55 pediatric OSA patients treated with AT	5–12 y	Taiwan, China	Prospective pre-AT, 3-month and 12-month follow-up; mild AHI ≥2 to < 5, moderate ≥5 to < 10, severe ≥ 10	Illumina MiSeq 2 × 300; V3–V4; primers 341F/806R; FLASH 1.2.11, QIIME 1.9.1, UCHIME, USEARCH 7.0.1090, Silva 123, PyNAST, Tax4Fun/KEGG, LEfSe	Sampling/storage: Morning stool; snap-frozen and stored at −80 °C; baseline, 3 and 12 months; Obesity: BMI recorded; obesity/weight effects considered but healthy controls absent; Diet: Short Food Frequency Questionnaire used; Antibiotic/probiotic: Stool collection postponed ≥2 weeks after antibiotic treatment; postoperative antibiotic use incompletely captured
Adult human clinical/cohort studies						
Ko et al. (2019)	113 (93 OSA)	Adults; mean 39–49 y	China	PSG; mild 5 < AHI ≤ 15 (*n* = 40), moderate 15 < AHI ≤ 30 (*n* = 23), severe AHI≥30 (*n* = 30)	Illumina MiSeq 2 × 300; V3–V4; primers ACTCCTACGGGAGGCAGCAG/GGACTACHVGGGTWTCTAAT; QIIME, PICRUSt/KEGG	Sampling/storage: Fresh feces in Microbiome Test Kit; fasting blood/feces collected next morning; Obesity: BMI/waist higher in severe OSAHS; obesity/metabolic comorbidities not excluded; Diet: Diet not controlled; enterotype differences considered; Antibiotic/probiotic: NR
Li et al. (2023a)	48 (37 OSA)	Adults, 18–65 y	China	Prospective case-control; PSG; no OSA AHI < 5, mild 5 < = AHI < 15, moderate 15 < = AHI < 30, severe AHI>=30; 31% severe OSA	Illumina MiSeq PE300; 16S rDNA V3–V4; Silva v128 OTUs at 97%; LEfSe, PCoA, Cytoscape; SPSS/R	Sampling/storage: 1 g fresh feces after overnight PSG and fasting; stored at −80 °C; fasting blood collected; Obesity: BMI higher in OSA/severe OSA; age not significantly different; regression adjusted for age, sex, BMI; Diet: Non-vegetarian; traditional Chinese food; detailed diet NR; Antibiotic/probiotic: Excluded antibiotics or microbial modifiers within 2 months; CPAP and surgery excluded
Li et al. (2021)	76 (55 OSA)	Adults; exact age NR	China	Human snoring/non-snoring cohort; PSG; OSA defined by AHI>=5; intestinal barrier biomarker study rather than microbiome-composition study	No microbiome sequencing; NA; D-LA by colorimetry and I-FABP by ELISA	Sampling/storage: Blood/plasma sampling; stool microbiome not analyzed; Obesity: BMI and OSA severity considered; details NR; Diet: NR; Antibiotic/probiotic: NR
Liu et al. (2024a)	37 (27 OSAHS; 10 controls)	Adults, 18–75 y; mean about 40–42 y	China	PSG; control *n* = 10, mild OSAHS *n* = 10, moderate-to-severe OSAHS *n* = 17	mNGS; platform NR; Metagenomic next-generation sequencing; species-level abundance; correlation/regression analyses	Sampling/storage: ~1 g morning feces after PSG; sterile dry tube; ice transport within 8 h; stored at −80 °C; Obesity: BMI comparable across groups; BMI correlated with F. prausnitzii; Diet: NR; Antibiotic/probiotic: Excluded antibiotics within 3 months and probiotics within 2 weeks
Animal/experimental model studies						
O'Connor et al. (2020)	Animal model: adult male rats	Adult	Ireland/UK; country NR in table source	Gut microbiota manipulation using broad-spectrum antibiotics; ventilatory responses to hypercapnia assessed; not an OSA cohort	16S rRNA sequencing; platform NR; 16S rRNA microbiome profiling; detailed region/pipeline NR	Sampling/storage: Fecal/intestinal microbiota samples; storage NR; Obesity: NA; Diet: Standard laboratory diet NR; Antibiotic/probiotic: Antibiotic cocktail used experimentally
Badran et al. (2020)	Animal model: male C57BL/6J mice; IH donors and naive recipients	8 wk	USA	OSA-like chronic intermittent hypoxia (IH) donor exposure for 6 wk; fecal microbiota transplantation into naive mice for 3 wk; sleep recorded	Illumina MiSeq; 16S rRNA V4; U515F/806R primers; FLASH, Cutadapt, SILVA v128	Sampling/storage: Fecal pellets processed with PowerFecal kit; storage NR; Obesity: NA; Diet: Standard chow NR; Antibiotic/probiotic: FMT intervention; no probiotic unless experimental protocol specified
Badran et al. (2023)	Animal model: male C57BL/6J mice; RA/IH FMT and IH/probiotic groups	8 wk	USA	Murine sleep-apnea model; room air vs. chronic IH donor microbiota transferred to naive mice; VSL#3 probiotic tested	16S rRNA amplicon sequencing; platform NR; 16S rRNA; detailed region/pipeline NR	Sampling/storage: Fecal samples from donor/recipient mice; storage NR; Obesity: NA; Diet: Standard chow NR; Antibiotic/probiotic: FMT and probiotic VSL#3 used experimentally
Moreno-Indias et al. (2016)	Animal model: C57BL/6 mice; IH and normoxic-recovery groups	NR	Spain/USA	OSA-like IH followed by normoxic recovery; endotoxemia and gut dysbiosis assessed	454 pyrosequencing; 16S rRNA; QIIME; exact region NR	Sampling/storage: Fecal samples; storage NR; Obesity: NA; Diet: NR; Antibiotic/probiotic: NR
Zhang et al. (2022)	Animal model: mice	NR	China	Chronic intermittent hypoxia model simulating OSA; systemic inflammation and metabolic dysfunction assessed	16S rRNA sequencing; platform NR; 16S rRNA; detailed region/pipeline NR	Sampling/storage: Fecal samples; storage NR; Obesity: NA; Diet: NR; Antibiotic/probiotic: NR
Li and Shi (2023)	Animal model: rats	NR	China	CIH-induced rats; daily 8 h IH with O_2_ falling from 21% to 8.5%; inflammatory markers, soft-palate histology and metabolomics assessed	Accu16S assay; UPLC-MS metabolomics; 16S-based fecal microbial profiling; detailed region/pipeline NR	Sampling/storage: Fecal samples for microbiota; serum and soft-palate tissue for metabolomics; storage NR; Obesity: NA; Diet: NR; Antibiotic/probiotic: NR
Dong et al. (2024)	Animal model: 90 male Sprague-Dawley rats; DIO subgroup n=15/group	2 wk at purchase; exposed after HFD modeling	China	Diet-induced obese rats; normoxia, IH (6% O_2_, 30 cycles/h, 8 h/day, 4 wk) or hypoxia/reoxygenation	Illumina HiSeq; 16S rRNA V4; primers 515F/806R; bioinformatics pipeline NR	Sampling/storage: Fecal samples after modeling/exposure; storage NR; Obesity: High-fat diet-induced obesity model; Diet: 8-wk high-fat diet before randomization; Antibiotic/probiotic: NR
Li et al. (2023b)	Animal model: C57BL/6J mice; Caco-2 cells also used	NR	China	Chronic intermittent hypoxia model with melatonin intervention; intestinal barrier and Th17 polarization assessed	High-throughput 16S rRNA sequencing; platform NR; 16S rRNA; detailed region/pipeline NR	Sampling/storage: Fecal/intestinal samples; storage NR; Obesity: NA; Diet: NR; Antibiotic/probiotic: Melatonin intervention; antibiotics/probiotics NR
Xue et al. (2025)	Animal model: C57BL/6J mice	NR	China/NR	CIH for 10 wk with inulin intervention; antibiotic intervention used to test microbiota contribution	16S rRNA sequencing; platform NR; 16S rRNA; detailed region/pipeline NR	Sampling/storage: Fecal/intestinal samples; storage NR; Obesity: NA; Diet: Inulin intervention; basal diet NR; Antibiotic/probiotic: Antibiotic intervention used experimentally
Poroyko et al. (2016)	Animal model: adult male C57BL/6J mice; SF *n* = 30, control *n* = 30; GF conventionalization subset	8 wk	USA/Spain	Chronic sleep fragmentation for 4 wk, with recovery subset; germ-free mice conventionalized with SF or control microbiota	Illumina MiSeq; 16S rRNA V4; RDP v9; Mothur; Metastats, PCoA/ANOSIM	Sampling/storage: Stool collected every other day for 42 d in subset; fecal/cecal material used for microbiota transfer; Obesity: NA; Diet: Normal chow; no high-fat or transgenic model; Antibiotic/probiotic: FMT/conventionalization used experimentally
Wang et al. (2022)	Animal model: 72 male C57BL/6J mice	6 wk at purchase; randomized at 8 wk	China	Eight groups: normoxia, CIH, HFD, CIH+HFD, normal sleep, CSF, NS+HFD, CSF+HFD; 10-wk intervention	Illumina MiSeq; 16S rRNA V3–V4; QIIME; OTUs at 97%; PICRUSt; serum metabolomics	Sampling/storage: Colon contents collected the morning after intervention; stored for 16S analysis; Obesity: High-fat diet arms included; Diet: Chow or high-fat diet; Antibiotic/probiotic: NR
Triplett et al. (2020)	Animal model: Sprague-Dawley rats	NR	USA	Acute/chronic sleep fragmentation; temporal and gut-region-specific microbiota and intestinal morphology assessed	16S rRNA sequencing; platform NR; 16S rRNA; detailed region/pipeline NR	Sampling/storage: Luminal/mucosal gut samples by region; storage NR; Obesity: NA; Diet: NR; Antibiotic/probiotic: NR
Sanford et al. (2021)	Animal model: male C57BL/6NCrl mice	NR	USA	Sleep fragmentation during light period vs. dark-period disturbance vs. home-cage control; 10-d exposure plus recovery/rechallenge design	Illumina MiSeq; 16S rRNA; Illumina 16S metagenomics protocol; Illumina Metagenomics software; dChip	Sampling/storage: Fecal samples at light onset, midday, light offset and midnight; repeated after recovery; Obesity: NA; Diet: Standard lab chow; Antibiotic/probiotic: NR

NR, not reported; NA, not applicable; SDB, sleep-disordered breathing; OSA/OSAHS/OSAS, obstructive sleep apnea/hypopnea syndrome; PSG, polysomnography; AHI/OAHI, apnea-hypopnea index/obstructive apnea-hypopnea index; SCR/SCS, Sleep Clinical Record/Score; F/B, Firmicutes/Bacteroidetes ratio; SCFA, short-chain fatty acid; HCY, homocysteine; AT, adenotonsillectomy; OWO, overweight/obesity; NW, normal weight; IH/CIH, intermittent/chronic intermittent hypoxia; SF/CSF, sleep fragmentation/chronic sleep fragmentation; SD, sleep deprivation; DIO, diet-induced obesity; HFD, high-fat diet; RA, room air; FMT, fecal microbiota transplantation; mNGS, metagenomic next-generation sequencing. For animal studies, the species or strain is stated in the Participants/animal species column where available.

### 4.1 Endocrine pathway

The HPA axis is a central neuroendocrine stress system involved in sleep regulation and is considered a potential contributor to SDB-related neurobehavioral phenotypes. Under physiological conditions, slow-wave sleep inhibits HPA-axis activity, whereas SF and repeated arousals may disrupt this regulatory rhythm, leading to abnormal glucocorticoid secretion patterns (O'byrne et al., 2021; De Feijter et al., 2022). In pediatric SDB, existing studies suggest alterations in HPA-axis-related markers, although findings remain inconsistent. A meta-analysis found that morning salivary cortisol levels were lower in children with OSA than in healthy controls. This reduced morning cortisol level in children has been interpreted as a possible reflection of HPA axis hyporesponsiveness following chronic psychophysiological stress (Imani et al., 2020). Gut microbiota dysbiosis may influence brain development and cognitive-behavioral outcomes through the HPA axis. Experimental studies in mice have shown that the absence of gut microbiota leads to increased corticotropin-releasing hormone (CRH) mRNA expression and elevated corticosterone levels. Increased basal HPA axis activity and exaggerated stress responses may contribute to bidirectional dysregulation between the hypothalamus and hippocampus (Sudo et al., 2004). Cortisol can affect intestinal permeability, transit time, and microbial composition through actions on intestinal epithelial cells, immune cells, enteroendocrine cells, and the ENS (Rusch et al., 2023; Karakan et al., 2021). It can also act on the hippocampus, amygdala, and prefrontal cortex, thereby influencing learning, memory, emotional regulation, and executive function (Rusch et al., 2023; Sudo et al., 2004). Animal studies further indicate that the gut microbiota can regulate the circadian rhythm and stress responsiveness of the HPA axis, and that fecal microbiota transplantation (FMT) and *Limosilactobacillus reuteri* may partially restore glucocorticoid rhythmicity (Tofani et al., 2025). SF and IH may therefore affect attention, executive control, and emotional regulation by altering HPA axis rhythmicity.

### 4.2 Neural pathway

#### 4.2.1 Vagus nerve, ENS and brainstem signaling

The ENS and vagus nerve constitute a relatively direct neural communication pathway within the MGBA. The vagus nerve is composed predominantly of afferent fibers, which can transmit signals derived from intestinal nutrients, cytokines, gut hormones, microbial metabolites, and neurotransmitters to the nucleus tractus solitarius (NTS) in the brainstem. These signals may further influence central networks involved in autonomic regulation, stress responses, sleep-wake states, and emotional behavior (Berthoud and Neuhuber, 2000; Fülling et al., 2019; Nøhr et al., 2015). Therefore, from anatomical and physiological perspectives, the ENS-vagus nerve pathway may provide a potential route through which the gut microbiota influences brain function.

Animal studies provide relatively strong mechanistic support for this pathway. Mouse studies indicate that administration of *Lactobacillus rhamnosus* modulates gamma-aminobutyric acid (GABA) receptor expression in the brain and alleviates anxiety and depression-related behaviors. In the vagus nerve section (VNX) group, mice exhibited both reversal of behavioral improvements and loss of GABA receptor upregulation and c-Fos neuronal activation (Bravo et al., 2011; Bharwani et al., 2020). *Lactobacillus rhamnosus* JB-1 induces vagal activity by modulating intrinsic pro-afferent neurons within the ENS (Perez-Burgos et al., 2013), indicating vagus-mediated communication between gut microbiota and the brain. In a mouse model of autism spectrum disorder, *Lactobacillus* restored social interaction-induced synaptic plasticity in the ventral tegmental area via a vagus nerve-dependent mechanism (Sgritta et al., 2019). Further research indicates that *Campylobacter jejuni* infection in mice enhances anxiety-like behavior, potentially via vagus nerve-mediated neural activation in the NTS, paraventricular nucleus, periventricular nucleus, and amygdala (Gaykema et al., 2004; Goehler et al., 2008). Furthermore, the research by Bercik et al. demonstrated that the anxiety-like behavior mediated by the vagus nerve in mice had its anti-anxiety benefits eliminated in mice after VNX. Because *Bacillus lonicella* reduces the excitability of intestinal neurons, it may signal the central nervous system by activating the vagus nerve pathway at the level of the intestinal nervous system (Bercik et al., 2011). Moreover, the vagus nerve is implicated in neurodegenerative disease progression, with reduced subsequent Parkinson's disease risk observed in young patients undergoing total vagotomy (Svensson et al., 2015). These results suggest that there is a functional connection between the gut microbiota, ENS and the vagus nerve, but this evidence mainly comes from animal models and other neurobehavioral disease models.

Furthermore, research suggests vagal afferents respond to numerous intestinal stimuli, including nutrients, cytokines, gut hormones, metabolites, and neurotransmitters (Nøhr et al., 2015). Inflammatory signals from the gut may also be transmitted to the brain via the vagus nerve. Rao and colleagues found that gut microbiota can regulate IL-1β-mediated hypothalamic pathways via this nerve (Rao et al., 2017). Moreover, long-term vagus nerve stimulation can trigger changes in the levels of multiple neurotransmitters, including GABA, 5-hydroxytryptamine (5-HT), and norepinephrine, thereby exerting additional regulatory effects on sleep (Ressler and Mayberg, 2007). This suggests that the gut microbiota and its metabolites may directly or indirectly act upon the central nervous system via the ENS, thereby influencing cognitive behavioral changes, with the enteric nerve-vagus nerve pathway playing a pivotal role. Pediatric SDB may be accompanied by IH, SF, autonomic imbalance, and abnormalities in brainstem-related regulation. These factors may theoretically alter intestinal motility, secretion, permeability, and microbial composition, and may further influence sleep regulation, attention, emotional responses, and executive function through vagal afferent signaling.

#### 4.2.2 Neurotransmitters

Tryptophan is an important precursor of 5-HT, melatonin, kynurenine and many indole metabolites (Liu et al., 2021). Disrupted microbiota may impair tryptophan availability and enterochromaffin cell function (Yano et al., 2015), thereby altering peripheral 5-HT synthesis and the metabolic flow of tryptophan. 5-HT is closely associated with mood regulation, impulse control, and sleep-wake rhythms. Direct evidence indicates that, in pediatric SDB patients with cognitive impairment, reduced relative abundance of *Clostridium* species in gut microbiota is associated with impaired tryptophan absorption (Tang et al., 2024). This finding provides preliminary evidence for a potential link among pediatric SDB, gut microbiota alterations, tryptophan metabolism, and cognitive function. In addition, evidence from pediatric neurodevelopmental disorders may provide indirect support for the view that tryptophan metabolism is a gut-brain axis pathway worthy of attention in pediatric neurobehavioral diseases. *Bacteroides* can convert pristine tryptophan into multiple indole metabolites, such as 5-HT, melatonin, and indoleacetaldehyde. Research has found significantly reduced indoleacetaldehyde levels in children with autism spectrum disorder (ASD) compared to controls (Dan et al., 2020). In addition, the decline of *Bacteroides* in the ASD group between 2 and 3 years old was negatively correlated with indoleacetaldehyde, and the abnormal tryptophan metabolism in some children with ASD may be related to the imbalance of gut microbiota (Dan et al., 2020). The 5-hydroxytryptamine 1A receptor (5-HT1AR) in the SD group of mice was significantly down-regulated in the hippocampus (Yang et al., 2023).

GABA is the principal inhibitory neurotransmitter in the CNS and is also associated with sleep stability, anxiety-like behavior, and cognitive function. Certain gut bacteria, including Lactobacillus and *Bifidobacterium*, can produce or regulate GABA through the glutamate decarboxylase pathway, suggesting that the gut microbiota may influence GABA signaling, sleep, and emotional regulation (Otaru et al., 2021; Liu et al., 2024b). Intervention studies have shown that specific strains, including *Limosilactobacillus reuteri* PBS072 and *Bifidobacterium* breve, elevated GABA levels and improved cognitive functions including short-term memory, attention, and executive function in subjects (Nobile et al., 2022). Elevated urinary GABA levels in children with cognitive impairment and OSA suggests overall elevated systemic GABA levels in pediatric OSA patients with cognitive dysfunction (Ji et al., 2025). This suggests that abnormalities in GABA metabolism may be associated with cognitive phenotypes related to pediatric OSA.

Melatonin is an important sleep rhythm-regulating molecule related to tryptophan metabolism and also has antioxidant and anti-inflammatory properties. Animal studies have shown that melatonin supplementation alleviated IH-induced intestinal mucosal injury, gut microbiota dysbiosis, intestinal Th17 polarization, and systemic low-grade inflammatory response (Li et al., 2023b).

Tryptophan/5-HT, GABA, and melatonin-related pathways provide a biologically plausible framework for explaining the link between gut microbiota dysbiosis and cognitive-behavioral abnormalities in pediatric SDB. Existing pediatric SDB evidence mainly focuses on preliminary associations involving gut microbiota alterations, tryptophan-related metabolic abnormalities, and urinary GABA changes, whereas animal studies and studies of other pediatric neurodevelopmental disorders provide mechanistic support.

### 4.3 Immune-inflammatory pathway

The immune-inflammatory pathway is one potential mechanism linking pediatric SDB, gut microbiota dysbiosis, and cognitive-behavioral abnormalities. In terms of direct evidence from pediatric OSA, Huang et al. (2024) found that gut microbiota β-diversity and specific marker taxa were associated with blood pressure changes in children with OSA. In particular, *Acinetobacter* was associated with IL-17, TNF-α, and blood pressure fluctuations, whereas IL-8 and C-C motif chemokine ligand 5 (CCL5) showed stronger associations with improvements in OSA severity. As two critical indicators, TNF-α and IL-6 are closely related to the occurrence and progression of OSA (Kheirandish-Gozal and Gozal, 2019; Ko et al., 2019; Wali et al., 2021), which are associated with neurocognitive impairment (Tegeler et al., 2016; Fernandes et al., 2024). Pediatric OSA studies have suggested that inflammatory cytokines, such as IL-17 and TNF-α, may be involved in disease-related complications. FMT from IH mice induces inflammation and neurobehavioral deficits in newborn mice (Yan et al., 2024). Animal models provide further mechanistic support for this pathway. SF suppresses the inflammatory chemokines CCL3 and CCL4 and their receptor C-C chemokine receptor type 5 (CCR5) in male C57BL/6NCrl mice, while maintaining C-X-C motif chemokine ligand 13 (Cxcl13), which maintains the immunomucosal barrier. Concurrently, elevated cortical cytokine IL-33 suggests neuroinflammation. These data indicate that SF alters the microbiome and suppresses mucosal immunity, while brain inflammatory mediators are upregulated (Sanford et al., 2021). Interventional animal studies have shown that salvia, a traditional Chinese medicine and functional tea widely used to protect learning and memory capacity, reduces systemic inflammation by decreasing gut inflammation and barrier disruption, subsequently lowering blood-brain barrier and neuronal damage, and further diminishing neuroinflammation in the hippocampus (Zhang et al., 2025). These findings provide additional convincing support that imbalance of the gut microbiota contributes at least partly to the development of cognitive impairment.

### 4.4 Metabolic pathway

Short-chain fatty acids (SCFAs) are mainly produced through the fermentation of dietary fiber by gut microbiota and include acetate, propionate, and butyrate. Several SCFA-producing taxa, such as *Faecalibacterium, Roseburia, Bifidobacterium, Ruminococcus*, and some *Bacteroides* species, are considered to be involved in maintaining intestinal barrier integrity, immune homeostasis, and anti-inflammatory responses (Den Besten et al., 2013; Lenoir et al., 2020; Nie et al., 2021). Some SCFAs can penetrate the blood-brain barrier, thereby shaping microglial growth and development, governing their activation and maturation, and boosting the brain's immune and immunological defense functions (Lv et al., 2023). Bacteria producing SCFAs are reduced in OSA patients (Ko et al., 2019), potentially due to altered gut microbiota. SCFAs are primarily produced by beneficial bacteria including Lactobacillaceae, Ruminococcaceae, Erysipelotrichaceae, Bifidobacteriaceae, and *Clostridium* (Den Besten et al., 2013). In terms of direct evidence, studies on gut microbiota alterations in pediatric OSA patients have confirmed reduced abundance of SCFA-producing bacteria or increased abundance of pathogenic bacteria. In pediatric OSA patients, SCFA-producing genera such as *Bacteroides, Bifidobacterium, Ruminococcus, Collinsella*, and *Faecalibacterium* exhibit a declining trend (Valentini et al., 2020; Lenoir et al., 2020; Nie et al., 2021). Adult OSA studies provide indirect support for this pathway. Ko et al. found that some SCFA-producing taxa were reduced in patients with OSA and were associated with metabolic abnormalities (Ko et al., 2019). Animal studies provide stronger mechanistic support for the SCFA pathway. In a sleep-deprivation mouse model, butyrate intervention significantly ameliorated intestinal mucosal injury and inflammatory responses by inhibiting the histone deacetylase 3 (HDAC3)-glycogen synthase kinase-3 beta (GSK-3β)-nuclear factor erythroid 2-related factor 2 (Nrf2)-NF-κB signaling cascade (Gao et al., 2023b). Furthermore, butyrate esters were identified as the effector responsible for Dact3-modulated effects of *F. prausnitzii*. The gut microbiota directly induces Dact3 upregulation via Wnt/JNK-associated pathways, providing novel insights into host molecular mechanisms involved in the anti-inflammatory effects of the beneficial commensal bacterium *F. prausnitzii* (Lenoir et al., 2020). Conversely, SCFAs such as butyrate promote proliferation and repair of intestinal epithelial cells, improve gut microbiota homeostasis, and suppress inflammatory responses (Gao et al., 2023a).

In addition to SCFAs, bile acid and fatty acid metabolism may also be involved in SDB-related pathological processes. Microbiome alterations induced by IH exposure primarily impact bile acid and fatty acid metabolism (Zhang et al., 2022). Research indicates that dysbiosis leads to a deficiency in secondary bile acids (SBA), thereby promoting a pro-inflammatory state within the gut, which can be treated through SBA restoration (Sinha et al., 2020). In a chronic hypoxia animal model, reduced abundances of beneficial gut microbes, including *Bacteroides thetaiotaomicron* and *Parabacteroides distasonis*, and accumulation of microbiota-derived cholic acid were observed. These changes were accompanied by white matter injury, impaired brain maturation, and neuroinflammatory alterations. Supplementation with *B. thetaiotaomicron* and *P. distasonis* restored cholic acid concentrations, rescuing leukocyte immunity and inflammation induced by IH. Gut microbiota-derived bile acids mediate neonatal leukocyte immunity and cerebral inflammation, leading to brain immaturity under IH (Yan et al., 2024). In addition, *Ruminococcus torques* alleviates intestinal diseases and gut barrier dysfunction by regulating the gut microbiota and bile acid metabolism (Lou et al., 2025). Together, these findings suggest that bile acid metabolism may link hypoxia, gut microbiota alterations, and neuroinflammation.

SCFAs, bile acids, and fatty acid metabolism provide a biologically plausible metabolic framework for explaining relationships among gut microbiota dysbiosis, inflammatory responses, and cognitive-behavioral abnormalities in pediatric SDB. Existing pediatric evidence mainly supports an association between OSA and alterations in some SCFA-producing taxa, whereas adult OSA studies and animal models suggest that SCFAs, bile acids, and fatty acid metabolism may influence brain function through intestinal barrier regulation, systemic inflammation, blood-brain barrier integrity, and neuroinflammation. Compared with taxonomic profiling, microbiota-derived metabolites such as SCFAs, bile acids, and tryptophan metabolites may provide more functionally stable and mechanistically proximal biomarkers, and therefore hold greater potential for translational diagnostic applications.

## 5 Clinical implications and future directions

Given the potential interaction between the gut microbiota and pediatric SDB, interventions such as probiotics, prebiotics, synbiotics, postbiotics, dietary fiber, dietary pattern modification, and FMT have biological plausibility. Their potential effects may be related to neurotransmitters and gut microbiota-derived metabolites. However, evidence supporting these interventions in pediatric SDB remains insufficient. Therefore, they should be positioned as future research directions rather than established clinical treatments.

### 5.1 Probiotic therapy

Adult clinical studies and animal experiments suggest that specific probiotics can increase SCFA production, improve intestinal barrier function, reduce inflammatory cytokine levels, and influence sleep and neurobehavioral phenotypes by modulating GABA, 5-HT, tryptophan metabolism, and vagus nerve-related signaling. However, no definitive studies have yet been conducted in pediatric OSA. Huang et al. (2024) found that changes in genera such as Veillonella and Agathobacter were associated with improvements in emotional distress. Targeting *Veillonella, Bifidobacterium*, and *Flavonifractor* may therefore represent a potential future adjunctive approach to conventional OSA treatment. Badran et al. (2023) found in a C57BL/6J mouse model of sleep apnea-like IH that fecal microbiota transplantation from IH-exposed donors induced elevated blood pressure, increased trimethylamine N-oxide (TMAO), and impaired aortic and coronary artery function in recipient mice. Administration of the probiotic VSL#3 attenuated some of the FMT-induced vascular abnormalities. Notably, in mice directly exposed to IH, VSL#3 did not sufficiently reduce blood pressure or prevent coronary artery dysfunction. This study supports the involvement of the gut microbiota in OSA-related vascular injury. Randomized controlled trials in pediatric SDB are still needed to determine whether probiotics can improve PSG parameters, residual OSA, blood pressure, metabolic abnormalities, or cognitive and behavioral outcomes.

### 5.2 Prebiotics, dietary fiber, and postbiotics

Currently, there are no definitive studies on the application of prebiotics, dietary fiber, or postbiotics in pediatric OSA. Xue et al. found that, in mice exposed to chronic IH, inulin alleviated IH-induced intestinal barrier dysfunction by modulating the gut microbiota, restoring tight junction protein expression, and reducing systemic inflammation (Xue et al., 2025). This study provides mechanistic support for prebiotic intervention in OSA-related intestinal barrier injury. Future studies should evaluate whether high-fiber diets or standardized supplementation with prebiotics or postbiotics can improve intestinal barrier biomarkers, SCFA levels, inflammatory status, and the residual risk after adenotonsillectomy.

### 5.3 Dietary pattern modification and metabolic improvement

Dietary intervention should be regarded as an important component of microbiota modulation, but its effects cannot be fully separated from weight control, energy intake, and metabolic improvement. Pediatric SDB often coexists with obesity, poor dietary quality, disrupted sleep rhythms, and inflammation. Therefore, dietary improvement may act simultaneously through weight reduction, attenuation of systemic inflammation, metabolic improvement, and modulation of the gut microbiota. Chuang et al. (2023) reported dietary profiles of pediatric OSA patients, the effects of routine educational counseling, and predictors of clinical outcomes, suggesting that dietary behavior may contribute to recovery after pediatric OSA treatment. Huang et al. (2024) further found that improvement in emotional distress after OSA treatment in children was associated with changes in the gut microbiota. These studies suggest that diet and the gut microbiota may jointly influence symptoms and neurobehavioral outcomes in pediatric OSA.

### 5.4 Conventional OSA treatment and gut microbiota restoration

Adenotonsillectomy is a first-line treatment for pediatric OSA. Chuang et al. conducted a longitudinal study in 55 children aged 5–12 years with OSA, with follow-up before adenotonsillectomy and at 3 and 12 months after surgery. They found that PSG parameters improved after adenotonsillectomy, accompanied by changes in α-diversity, β-diversity, specific microbial taxa, and predicted metabolic pathways (Chuang et al., 2024). Huang et al. also observed changes in symptoms, emotional distress, and gut microbiota diversity indices after OSA treatment in children (Huang et al., 2024). These findings suggest that relief of upper airway obstruction may be accompanied by restoration of the gut ecosystem. However, because of the lack of healthy longitudinal controls and insufficient control for postoperative antibiotic exposure and dietary changes, it remains impossible to prove that microbiota changes are directly caused by OSA remission itself. Direct evidence on the effects of pediatric continuous positive airway pressure (CPAP), weight control, and sleep improvement on the gut microbiota is still lacking.

### 5.5 FMT

FMT should not be proposed as a therapeutic strategy for pediatric SDB. Although FMT has pediatric evidence for selected conditions such as recurrent *Clostridioides difficile* infection (Tun et al., 2022), pediatric SDB is not a life-threatening microbiota-deficiency disorder, and its pathogenesis involves multiple factors, including adenotonsillar hypertrophy, craniofacial structure, obesity, neuromuscular tone, and sleep regulation. FMT also carries concerns regarding donor-derived pathogen transmission, transfer of antimicrobial resistance genes, uncertain long-term immune and metabolic effects, inadequate pediatric consent/assent capacity, and difficulty in monitoring delayed adverse events. Therefore, at the current stage of evidence, FMT should be discussed only as a theoretical concept or as a research tool under strict ethical approval, rather than as a treatment recommendation for pediatric SDB.

## 6 Conclusions and perspectives

Pediatric SDB is increasingly recognized as a systemic condition associated with alterations in gut microbiota composition, diversity, and metabolic activity. Current evidence suggests that these microbial changes are observed alongside IH, SF, metabolic dysregulation, and systemic inflammatory responses, indicating a potential link between respiratory sleep disorders and host-microbiome interactions. Based on the current literature, this review proposes a conceptual gut-brain-lung framework to explain the possible links among pediatric SDB, gut microbiota dysbiosis, and cognitive-behavioral alterations. SDB-related physiological stressors may contribute to intestinal microenvironment disturbances, while gut microbial alterations and their derived metabolites may be associated with systemic immune, metabolic, and neuroregulatory processes that are relevant to cognitive and behavioral outcomes.

However, direct pediatric evidence regarding the relationship between gut microbiota alterations and cognitive-behavioral outcomes in children with SDB remains limited. Most mechanistic evidence is derived from adult OSA studies, animal models, and broader research on MGBA-related disorders. Therefore, the microbiota-related pathways proposed in this review should be regarded as biologically plausible and hypothesis-generating rather than clinically established mechanisms in pediatric SDB. Future research urgently requires longitudinal cohort and interventional studies in pediatric populations to establish causal relationships between specific microbial profiles and impairments in particular cognitive domains. Furthermore, clinical feasibility studies should explore restoring the microbiota through prebiotic, probiotic, or dietary interventions to improve cognitive outcomes in children with SDB, thereby pioneering new pathways for personalized and comprehensive management of pediatric SDB.
